# (*E*)-*N*′-(2-Hydroxy­benzyl­idene)-3,4,5-trimethoxy­benzohydrazide

**DOI:** 10.1107/S1600536808006077

**Published:** 2008-05-07

**Authors:** Yu-Min Wang, Zhen-Dong Zhao, Yu-Xiang Chen, Liang-Wu Bi

**Affiliations:** a Institute of Chemical Industry of Forest Products, Chinese Academy of Forestry, Nanjing 210042, People’s Republic of China

## Abstract

The title compound, C_17_H_18_N_2_O_5_, was synthesized from 3,4,5-trimethoxy­benzohydrazide and 2-hydroxy­benzaldehyde. The dihedral angle between the planes of the two benzene rings is 29.9 (2)°. The crystal structure involves intra­molecular O—H⋯N, and inter­molecular N—H⋯O and C—H⋯O hydrogen bonds.

## Related literature

For related literature, see: Yang *et al.* (1996 ▶); Nawar *et al.* (2000 ▶). Gardner *et al.* (1991 ▶); Labouta *et al.* (1989 ▶).
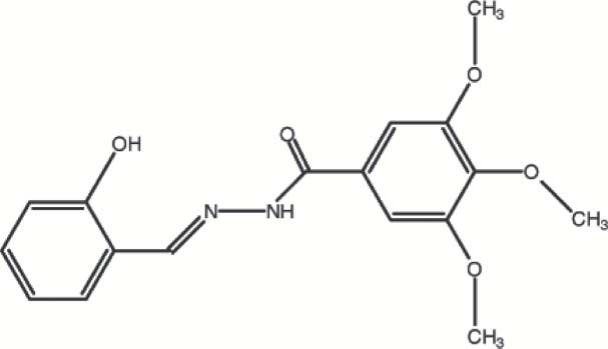


## Experimental

### 

#### Crystal data

C_17_H_18_N_2_O_5_
*M* *_r_* = 330.33
Monoclinic, 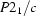

*a* = 15.348 (12) Å
*b* = 13.330 (11) Å
*c* = 8.299 (7) Å
β = 99.854 (16)°
*V* = 1673 (2) Å^3^
*Z* = 4
Mo *K*α radiation
μ = 0.10 mm^−1^
*T* = 273 (2) K
0.10 × 0.06 × 0.04 mm

#### Data collection

Bruker APEX CCD area-detector diffractometer
Absorption correction: multi-scan (*SADABS*; Sheldrick, 1996 ▶) *T*_min_ = 0.991, *T*_max_ = 0.995
8200 measured reflections
2952 independent reflections
1945 reflections with *I* > 2σ(*I*)
*R*_int_ = 0.048

#### Refinement

*R*[*F* ^2^ > 2σ(*F* ^2^)] = 0.068
*wR*(*F* ^2^) = 0.194
*S* = 1.00
2952 reflections
219 parameters
H-atom parameters constrained
Δρ_max_ = 0.59 e Å^−3^
Δρ_min_ = −0.26 e Å^−3^

### 

Data collection: *SMART* (Bruker, 1997 ▶); cell refinement: *SAINT* (Bruker, 1997 ▶); data reduction: *SAINT*; program(s) used to solve structure: *SHELXS97* (Sheldrick, 2008 ▶); program(s) used to refine structure: *SHELXL97* (Sheldrick, 2008 ▶); molecular graphics: *SHELXTL* (Sheldrick, 2008 ▶); software used to prepare material for publication: *SHELXTL*.

## Supplementary Material

Crystal structure: contains datablocks I, global. DOI: 10.1107/S1600536808006077/at2541sup1.cif

Structure factors: contains datablocks I. DOI: 10.1107/S1600536808006077/at2541Isup2.hkl

Additional supplementary materials:  crystallographic information; 3D view; checkCIF report

## Figures and Tables

**Table 1:** Hydrogen-bond geometry (Å, °)

*D*—H⋯*A*	*D*—H	H⋯*A*	*D*⋯*A*	*D*—H⋯*A*
O1—H1⋯N1	0.82	1.94	2.670 (4)	147
N2—H2⋯O2^i^	0.86	2.00	2.826 (4)	161 (1)
C7—H7⋯O2^i^	0.93	2.48	3.240 (5)	139

Symmetry code: (i) 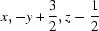.

**Fig. 1.**
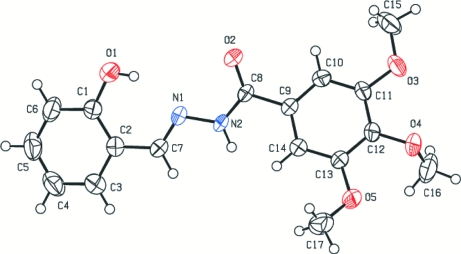
A view of the molecular structure of the title compound, showing displacement ellipsoids at the 50% probability level.

**Fig. 2.**
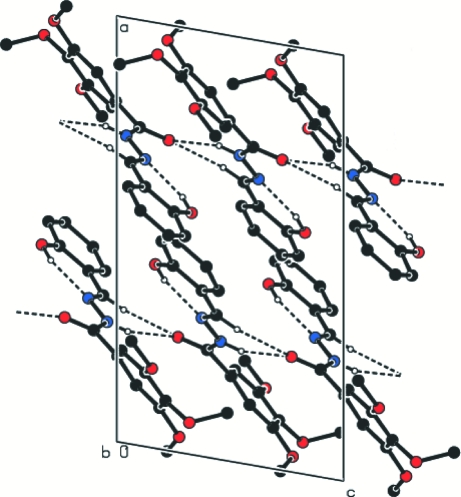
The packing diagram for (I) showing three dimensional network formed via hydrogen bonding.

## supplementary crystallographic information

### Comment

3,4,5-Trimethoxybenzohydrazide and their deviatives show moderate fungicidal and anti-bacterial activities (Gardner *et al.*,1991). The antibacterial activity of formylhydrazines and formylhydrazones has been reported by Labouta *et al.* (1989). Many derivatives of formylhydrazines have interesting biological properties. So we synthesized the title compound (I) and report here its crystal structure.

The molecular structure of (I) is shown in Fig. 1, where the dash lines indicate N–H···O and O—H···N hydrogen bonds (Table 2). The atoms C7, N1, N2, C8 and O2 almost share a same plane for its delocalized structure. The dihedral angle between the planes of the two phenyl rings is 29.9 (217)°.

In the crystal structure, there is a intramolecular O—H···N hydrogen bond and two intermolecular N—H···O and C—H···O hydrogen bonds.

### Experimental

An ethanol solution (50 ml) of 3,4,5-trimethoxybenzohydrazide (0.01 mol) and 2-hydroxybenzaldehyde (0.01 mol) was refluxed and stirred for 4 h. The mixture was cooled and the resulting solid product, (I), was collected by filtration. Crystals suitable for single-crystal X-ray diffraction were grown by slow evaporation of a solution in THF.

### Refinement

All H atoms bonded to the C atoms were placed geometrically at the distances of 0.93–0.96 Å and included in the refinement in riding motion approximation with *U*_iso_(H) = 1.2 or 1.5*U*_eq_ of the carrier atom.

### Crystal data

**Table:**

C_17_H_18_N_2_O_5_	*F*_000_ = 696
*M**_r_* = 330.33	*D*_x_ = 1.312 Mg m^−^^3^
Monoclinic, *P*2_1_/*c*	Mo *K*α radiation λ = 0.71073 Å
Hall symbol: -P 2ybc	Cell parameters from 2179 reflections
*a* = 15.348 (12) Å	θ = 2.7–22.9º
*b* = 13.330 (11) Å	µ = 0.10 mm^−^^1^
*c* = 8.299 (7) Å	*T* = 273 (2) K
β = 99.854 (16)º	Needle, colourless
*V* = 1673 (2) Å^3^	0.10 × 0.06 × 0.04 mm
*Z* = 4	

### Data collection

**Table:**

Bruker APEX CCD area-detector diffractometer	2952 independent reflections
Radiation source: fine-focus sealed tube	1945 reflections with *I* > 2σ(*I*)
Monochromator: graphite	*R*_int_ = 0.048
*T* = 273(2) K	θ_max_ = 25.0º
φ and ω scans	θ_min_ = 1.4º
Absorption correction: multi-scan(SADABS; Sheldrick, 1996)	*h* = −14→18
*T*_min_ = 0.991, *T*_max_ = 0.995	*k* = −14→15
8200 measured reflections	*l* = −9→9

### Refinement

**Table:**

Refinement on *F*^2^	Hydrogen site location: inferred from neighbouring sites
Least-squares matrix: full	H-atom parameters constrained
*R*[*F*^2^ > 2σ(*F*^2^)] = 0.068	*w* = 1/[σ^2^(*F*_o_^2^) + (0.09*P*)^2^ + 1.3*P*] where *P* = (*F*_o_^2^ + 2*F*_c_^2^)/3
*wR*(*F*^2^) = 0.194	(Δ/σ)_max_ < 0.001
*S* = 1.00	Δρ_max_ = 0.59 e Å^−^^3^
2952 reflections	Δρ_min_ = −0.25 e Å^−^^3^
219 parameters	Extinction correction: SHELXL97 (Sheldrick, 2008), Fc^*^=kFc[1+0.001xFc^2^λ^3^/sin(2θ)]^-1/4^
Primary atom site location: structure-invariant direct methods	Extinction coefficient: 0.009 (2)
Secondary atom site location: difference Fourier map	

### Special details

**Table:**

Geometry. All e.s.d.'s (except the e.s.d. in the dihedral angle between two l.s. planes) are estimated using the full covariance matrix. The cell e.s.d.'s are taken into account individually in the estimation of e.s.d.'s in distances, angles and torsion angles; correlations between e.s.d.'s in cell parameters are only used when they are defined by crystal symmetry. An approximate (isotropic) treatment of cell e.s.d.'s is used for estimating e.s.d.'s involving l.s. planes.
Refinement. Refinement of *F*^2^ against ALL reflections. The weighted *R*-factor *wR* and goodness of fit *S* are based on *F*^2^, conventional *R*-factors *R* are based on *F*, with *F* set to zero for negative *F*^2^. The threshold expression of *F*^2^ > σ(*F*^2^) is used only for calculating *R*-factors(gt) *etc*. and is not relevant to the choice of reflections for refinement. *R*-factors based on *F*^2^ are statistically about twice as large as those based on *F*, and *R*- factors based on ALL data will be even larger.

### Fractional atomic coordinates and isotropic or equivalent isotropic displacement parameters (Å^2^)

**Table:**

	*x*	*y*	*z*	*U*_iso_*/*U*_eq_	
O1	0.56784 (18)	0.60473 (18)	0.8299 (3)	0.0620 (7)	
H1	0.6008	0.6424	0.7904	0.093*	
O2	0.72936 (16)	0.85318 (16)	0.7297 (3)	0.0523 (7)	
O3	0.81393 (18)	1.13644 (15)	0.3448 (4)	0.0684 (8)	
O4	0.92560 (17)	1.04300 (17)	0.1799 (3)	0.0613 (8)	
O5	0.96337 (16)	0.84707 (17)	0.2246 (3)	0.0586 (7)	
N1	0.67460 (17)	0.66256 (18)	0.6249 (3)	0.0390 (6)	
N2	0.72254 (17)	0.72742 (18)	0.5423 (3)	0.0412 (7)	
H2	0.7367	0.7084	0.4512	0.049*	
C1	0.5635 (2)	0.5152 (2)	0.7521 (4)	0.0445 (8)	
C2	0.6102 (2)	0.4977 (2)	0.6245 (4)	0.0423 (8)	
C3	0.6039 (2)	0.4020 (2)	0.5511 (4)	0.0541 (9)	
H3	0.6344	0.3888	0.4658	0.065*	
C4	0.5525 (3)	0.3270 (3)	0.6047 (5)	0.0643 (11)	
H4	0.5497	0.2636	0.5574	0.077*	
C5	0.5060 (3)	0.3479 (3)	0.7282 (5)	0.0651 (11)	
H5	0.4708	0.2983	0.7629	0.078*	
C6	0.5108 (3)	0.4403 (3)	0.8012 (4)	0.0602 (10)	
H6	0.4785	0.4530	0.8843	0.072*	
C7	0.6621 (2)	0.5750 (2)	0.5607 (4)	0.0421 (8)	
H7	0.6874	0.5602	0.4693	0.051*	
C8	0.7475 (2)	0.8195 (2)	0.6011 (4)	0.0379 (7)	
C9	0.7992 (2)	0.8781 (2)	0.4960 (4)	0.0368 (7)	
C10	0.7841 (2)	0.9807 (2)	0.4799 (4)	0.0441 (8)	
H10	0.7461	1.0123	0.5400	0.053*	
C11	0.8259 (2)	1.0356 (2)	0.3738 (4)	0.0461 (8)	
C12	0.8839 (2)	0.9881 (2)	0.2853 (4)	0.0460 (8)	
C13	0.9029 (2)	0.8862 (2)	0.3110 (4)	0.0428 (8)	
C14	0.8592 (2)	0.8308 (2)	0.4139 (4)	0.0415 (8)	
H14	0.8701	0.7624	0.4279	0.050*	
C15	0.7580 (3)	1.1888 (3)	0.4353 (6)	0.0807 (14)	
H15A	0.6995	1.1610	0.4119	0.121*	
H15B	0.7560	1.2584	0.4053	0.121*	
H15C	0.7806	1.1824	0.5501	0.121*	
C16	0.8877 (3)	1.0316 (3)	0.0131 (6)	0.0787 (14)	
H16A	0.8832	0.9615	−0.0138	0.118*	
H16B	0.9243	1.0644	−0.0538	0.118*	
H16C	0.8298	1.0611	−0.0062	0.118*	
C17	1.0023 (3)	0.7532 (3)	0.2783 (5)	0.0656 (11)	
H17A	1.0269	0.7572	0.3925	0.098*	
H17B	1.0483	0.7375	0.2171	0.098*	
H17C	0.9580	0.7017	0.2613	0.098*	

### Atomic displacement parameters (Å^2^)

**Table:**

	*U*^11^	*U*^22^	*U*^33^	*U*^12^	*U*^13^	*U*^23^
O1	0.085 (2)	0.0474 (14)	0.0639 (17)	−0.0132 (13)	0.0429 (14)	−0.0088 (12)
O2	0.0821 (17)	0.0435 (13)	0.0395 (14)	0.0001 (11)	0.0339 (12)	−0.0002 (10)
O3	0.0843 (19)	0.0262 (12)	0.108 (2)	0.0009 (11)	0.0555 (17)	0.0053 (12)
O4	0.0757 (18)	0.0431 (13)	0.077 (2)	−0.0130 (12)	0.0470 (15)	0.0080 (12)
O5	0.0677 (16)	0.0472 (14)	0.0734 (18)	0.0114 (12)	0.0475 (14)	0.0096 (12)
N1	0.0488 (16)	0.0375 (14)	0.0352 (15)	−0.0023 (11)	0.0199 (12)	0.0062 (11)
N2	0.0614 (17)	0.0367 (14)	0.0326 (15)	−0.0051 (12)	0.0283 (13)	0.0024 (11)
C1	0.059 (2)	0.0382 (17)	0.0386 (19)	−0.0069 (15)	0.0148 (16)	−0.0011 (14)
C2	0.0508 (19)	0.0364 (16)	0.0417 (19)	−0.0023 (14)	0.0135 (15)	0.0034 (13)
C3	0.071 (2)	0.0403 (18)	0.054 (2)	−0.0005 (17)	0.0179 (18)	−0.0032 (16)
C4	0.087 (3)	0.0363 (18)	0.066 (3)	−0.0093 (19)	0.006 (2)	−0.0028 (17)
C5	0.086 (3)	0.051 (2)	0.056 (2)	−0.029 (2)	0.009 (2)	0.0073 (18)
C6	0.077 (3)	0.062 (2)	0.046 (2)	−0.0220 (19)	0.0245 (19)	0.0042 (17)
C7	0.053 (2)	0.0424 (18)	0.0355 (18)	0.0009 (14)	0.0201 (15)	0.0022 (14)
C8	0.0511 (19)	0.0370 (16)	0.0299 (17)	0.0027 (14)	0.0188 (14)	0.0058 (13)
C9	0.0451 (18)	0.0351 (16)	0.0339 (17)	−0.0007 (13)	0.0169 (14)	0.0011 (12)
C10	0.051 (2)	0.0367 (17)	0.051 (2)	−0.0017 (14)	0.0265 (16)	−0.0048 (14)
C11	0.057 (2)	0.0260 (15)	0.061 (2)	−0.0032 (14)	0.0271 (17)	0.0020 (14)
C12	0.053 (2)	0.0320 (16)	0.060 (2)	−0.0071 (14)	0.0305 (17)	0.0021 (14)
C13	0.0484 (19)	0.0369 (17)	0.050 (2)	0.0004 (14)	0.0271 (16)	0.0015 (14)
C14	0.053 (2)	0.0328 (16)	0.0429 (19)	0.0012 (14)	0.0213 (15)	0.0014 (13)
C15	0.093 (3)	0.0329 (19)	0.128 (4)	0.0065 (19)	0.053 (3)	−0.003 (2)
C16	0.090 (3)	0.079 (3)	0.077 (3)	−0.001 (2)	0.044 (3)	0.033 (2)
C17	0.065 (2)	0.069 (2)	0.070 (3)	0.024 (2)	0.031 (2)	0.009 (2)

### Geometric parameters (Å, °)

**Table:**

O1—C1	1.353 (4)	C5—H5	0.9300
O1—H1	0.8200	C6—H6	0.9300
O2—C8	1.233 (3)	C7—H7	0.9300
O3—C11	1.373 (4)	C8—C9	1.496 (4)
O3—C15	1.417 (4)	C9—C14	1.387 (4)
O4—C12	1.379 (4)	C9—C10	1.390 (4)
O4—C16	1.415 (5)	C10—C11	1.384 (4)
O5—C13	1.369 (3)	C10—H10	0.9300
O5—C17	1.424 (4)	C11—C12	1.398 (4)
N1—C7	1.284 (4)	C12—C13	1.399 (4)
N1—N2	1.389 (3)	C13—C14	1.386 (4)
N2—C8	1.351 (4)	C14—H14	0.9300
N2—H2	0.8600	C15—H15A	0.9600
C1—C6	1.389 (5)	C15—H15B	0.9600
C1—C2	1.396 (4)	C15—H15C	0.9600
C2—C3	1.410 (5)	C16—H16A	0.9600
C2—C7	1.456 (4)	C16—H16B	0.9600
C3—C4	1.393 (5)	C16—H16C	0.9600
C3—H3	0.9300	C17—H17A	0.9600
C4—C5	1.373 (5)	C17—H17B	0.9600
C4—H4	0.9300	C17—H17C	0.9600
C5—C6	1.369 (5)		
			
C1—O1—H1	109.5	C10—C9—C8	118.3 (3)
C11—O3—C15	117.7 (3)	C11—C10—C9	119.6 (3)
C12—O4—C16	113.9 (3)	C11—C10—H10	120.2
C13—O5—C17	117.1 (3)	C9—C10—H10	120.2
C7—N1—N2	114.5 (2)	O3—C11—C10	124.5 (3)
C8—N2—N1	121.9 (2)	O3—C11—C12	115.4 (3)
C8—N2—H2	119.1	C10—C11—C12	120.1 (3)
N1—N2—H2	119.1	O4—C12—C11	120.0 (3)
O1—C1—C6	118.5 (3)	O4—C12—C13	120.3 (3)
O1—C1—C2	121.3 (3)	C11—C12—C13	119.6 (3)
C6—C1—C2	120.2 (3)	O5—C13—C14	124.3 (3)
C1—C2—C3	118.2 (3)	O5—C13—C12	115.6 (3)
C1—C2—C7	122.8 (3)	C14—C13—C12	120.1 (3)
C3—C2—C7	118.9 (3)	C13—C14—C9	119.5 (3)
C4—C3—C2	120.8 (3)	C13—C14—H14	120.2
C4—C3—H3	119.6	C9—C14—H14	120.2
C2—C3—H3	119.6	O3—C15—H15A	109.5
C5—C4—C3	119.2 (3)	O3—C15—H15B	109.5
C5—C4—H4	120.4	H15A—C15—H15B	109.5
C3—C4—H4	120.4	O3—C15—H15C	109.5
C6—C5—C4	121.2 (3)	H15A—C15—H15C	109.5
C6—C5—H5	119.4	H15B—C15—H15C	109.5
C4—C5—H5	119.4	O4—C16—H16A	109.5
C5—C6—C1	120.4 (3)	O4—C16—H16B	109.5
C5—C6—H6	119.8	H16A—C16—H16B	109.5
C1—C6—H6	119.8	O4—C16—H16C	109.5
N1—C7—C2	122.9 (3)	H16A—C16—H16C	109.5
N1—C7—H7	118.5	H16B—C16—H16C	109.5
C2—C7—H7	118.5	O5—C17—H17A	109.5
O2—C8—N2	123.5 (3)	O5—C17—H17B	109.5
O2—C8—C9	122.3 (3)	H17A—C17—H17B	109.5
N2—C8—C9	114.2 (2)	O5—C17—H17C	109.5
C14—C9—C10	120.9 (3)	H17A—C17—H17C	109.5
C14—C9—C8	120.8 (3)	H17B—C17—H17C	109.5
			
C7—N1—N2—C8	−174.6 (3)	C8—C9—C10—C11	−175.1 (3)
O1—C1—C2—C3	178.8 (3)	C15—O3—C11—C10	3.0 (6)
C6—C1—C2—C3	−1.5 (5)	C15—O3—C11—C12	−178.0 (3)
O1—C1—C2—C7	−3.6 (5)	C9—C10—C11—O3	178.1 (3)
C6—C1—C2—C7	176.1 (3)	C9—C10—C11—C12	−0.9 (5)
C1—C2—C3—C4	−0.1 (5)	C16—O4—C12—C11	−102.2 (4)
C7—C2—C3—C4	−177.8 (3)	C16—O4—C12—C13	81.3 (4)
C2—C3—C4—C5	1.5 (6)	O3—C11—C12—O4	1.1 (5)
C3—C4—C5—C6	−1.2 (6)	C10—C11—C12—O4	−179.9 (3)
C4—C5—C6—C1	−0.4 (6)	O3—C11—C12—C13	177.6 (3)
O1—C1—C6—C5	−178.5 (4)	C10—C11—C12—C13	−3.4 (5)
C2—C1—C6—C5	1.8 (6)	C17—O5—C13—C14	−18.9 (5)
N2—N1—C7—C2	−177.2 (3)	C17—O5—C13—C12	163.4 (3)
C1—C2—C7—N1	5.6 (5)	O4—C12—C13—O5	−0.7 (5)
C3—C2—C7—N1	−176.8 (3)	C11—C12—C13—O5	−177.1 (3)
N1—N2—C8—O2	−0.7 (5)	O4—C12—C13—C14	−178.4 (3)
N1—N2—C8—C9	179.5 (2)	C11—C12—C13—C14	5.1 (5)
O2—C8—C9—C14	143.6 (3)	O5—C13—C14—C9	179.9 (3)
N2—C8—C9—C14	−36.7 (4)	C12—C13—C14—C9	−2.5 (5)
O2—C8—C9—C10	−37.9 (4)	C10—C9—C14—C13	−1.8 (5)
N2—C8—C9—C10	141.9 (3)	C8—C9—C14—C13	176.8 (3)
C14—C9—C10—C11	3.5 (5)		

### Hydrogen-bond geometry (Å, °)

**Table:**

*D*—H···*A*	*D*—H	H···*A*	*D*···*A*	*D*—H···*A*
O1—H1···N1	0.82	1.94	2.670 (4)	147
N2—H2···O2^i^	0.86	2.00	2.826 (4)	161 (1)
C7—H7···O2^i^	0.93	2.48	3.240 (5)	139

Symmetry codes: (i) *x*, −*y*+3/2, *z*−1/2.
